# Combined Analysis of BSA-Seq and RNA-Seq Reveals Candidate Genes for *qGS1* Related to Sorghum Grain Size

**DOI:** 10.3390/plants14121791

**Published:** 2025-06-11

**Authors:** Qi Shen, Kai Wang, Lu Hu, Lei Li, Lihua Wang, Yongfei Wang, Yi-Hong Wang, Jieqin Li

**Affiliations:** 1 College of Agriculture, Anhui Science and Technology University, Fengyang 233100, China; qishen5552024@163.com (Q.S.); whlk523314@163.com (K.W.); m18256213879@163.com (L.H.); lilei200111@163.com (L.L.); wanglihuaerr@126.com (L.W.); wangyongfei@ahstu.edu.cn (Y.W.); 2 International Joint Research Center of Forage Bio-Breeding in Anhui Province, Chuzhou 233100, China; 3 Department of Biology, University of Louisiana at Lafayette, Lafayette, LA 70504, USA

**Keywords:** sorghum, grain size, BSA-Seq, RNA-Seq, candidate genes

## Abstract

Grain size is a crucial agronomic trait that significantly impacts yield potential in sorghum (*Sorghum bicolor*), making it a key focus for genetic improvement. In this study, we investigated the genetic basis of grain size variation using two contrasting sorghum accessions, PI302232 (small grain, Sg) and PI563512 (large grain, Lg). The 1000-grain weight, grain length, and grain width of Lg were 3.63-fold, 1.22-fold, and 1.65-fold higher than Sg, respectively. The 1000-grain weight in the F_2_ segregating population derived from the cross Sg and Lg parents exhibited the highest phenotypic variation and followed a normal distribution in the three traits. Using bulked segregant analysis sequencing (BSA-seq) with small- and large-grain bulks from the F_2_ population, two major quantitative trait loci (QTLs) for grain size were identified on chromosomes 1 and 3. Fine mapping with SSR markers narrowed the *qGS1* locus to a 1.03 Mb region on chromosome 1 (Chr01: 22,001,448–23,035,593), containing 49 candidate genes. To narrow down potential candidate genes, transcriptome analysis of spike tissues from Sg and Lg at 0 and 14 days after heading revealed 3719 differentially expressed genes (DEGs), primarily enriched in “starch and sucrose metabolism” and “phenylpropanoid biosynthesis” pathways. Integrating fine mapping intervals and RNA-seq data, 6 DEGs were identified within the *qGS1* region. Quantitative real-time PCR confirmed that 6 genes exhibited different expression at two stages. The expression and bioinformatics analysis showed Sobic.001G230700 was the most likely candidate gene for the qGS1 locus. This study provides new insights into the genetic regulation of grain size and a new target to improve grain size in sorghum.

## 1. Introduction

Sorghum [*Sorghum bicolor* (L) Moench] is an annual herbaceous plant in the genus Sorghum of the Poaceae family. It ranks fifth in global cereal crop production as it is nutritionally rich, serving as one of the traditional staple foods in China and for more than 500 million people in semi-arid regions of Africa and Southeast Asia [1]. Sorghum is also an efficient crop for brewing, feeding, fiber production, and bioenergy [2]. To date, food security in these regions remains a major challenge. Grain size is a crucial component of grain yield and one of the important research directions for enhancing sorghum yield [3]. Despite the versatility of sorghum grains, the genetic mechanisms underlying them remain unelucidated. Understanding the variation of sorghum grain size and its genetic basis will accelerate sorghum breeding in yield traits.

Grain formation is the result of synergistic interactions among multiple pathways, including hormonal signaling, transcriptional regulation, sugar metabolism (sucrose transport), and epigenetic regulation. In hormonal signaling, indole-3-acetic acid (IAA) regulates endosperm cellularization and grain initiation, while cytokinin (CK) promotes cell division and grain expansion [4]. Gibberellin (GA) enhances starch accumulation during grain filling, and abscisic acid (ABA) modulates grain maturation and dormancy [5]. Transcriptional regulation is also crucial, with MADS-box transcription factors governing endosperm development and grain size, and the *AP2/ERF* family regulating maize grain filling and nutrient transport [6]. Additionally, sugar metabolism, through “sucrose transporters and sugar signaling” pathways, controls grain filling by mediating carbohydrate allocation [7]. Finally, epigenetic regulation, including DNA methylation and histone modifications, influences grain size, as seen in wheat, where *TaABI3-A1* regulates starch and storage protein synthesis by epigenetically controlling grain quality during development [5,8,9].

In sorghum, grain size was influenced by multiple factors, including both genotype and environmental conditions [10,11]. While 186 QTLs associated with grain size have been identified across all 10 sorghum chromosomes, more than 80% of these are linked to 1000-grain weight [12]. Despite technological advancements and extensive research on grain size QTLs, fine-mapping and cloning of sorghum grain size genes remain relatively scarce. On chromosome 1, *qTGW1a* (*Sobic.001G341700*) has been cloned and identified as a homolog of the rice grain size gene *GS3*, and it negatively regulates grain size in sorghum [13]. The *qGW1* locus has been finely mapped to a 101 kb region on the short arm of chromosome 1, with *Sobic.001G038300* proposed as the candidate gene [14]. On chromosome 2, *qFHGS2.5* (*Sobic.002G216600*) has been identified as a homolog of the rice *DEP1* gene, which positively regulates grain number [15]. Additionally, *MGS1* has been discovered to control the multi-grain spikelet trait in sorghum, offering promising potential for yield improvement [16]. However, research on the cloning and identification of grain-related genes in sorghum lags behind that of other major cereal crops, such as rice [17], maize [18]. Sorghum has been widely considered a recalcitrant major crop for transformation, which limits the functional characterization of grain-related genes in sorghum [19].

In this study, we identified two sorghum accessions with distinct grain sizes: a large-grain accession (Lg, PI563512) and a small-grain accession (Sg, PI302232). An F_2_ population consisting of 402 recombinant individuals was developed using the two accessions as parents. Grain size traits were evaluated for the parents and F_2_ population. Bulked segregant analysis sequencing (BSA-seq) and RNA sequencing (RNA-seq) were employed to identify candidate genes associated with grain size. Our findings provide valuable germplasm resources and candidate genes for the genetic improvement of grain sorghum.

## 2. Materials and Methods

### 2.1. Plant Materials

The sorghum accessions Ls (PI563512) and Sg (PI302232) were provided by the Anhui Province International Joint Research Center of Forage Bio-breeding (Chuzhou, China). In this study, F_1_ was generated by crossing Sg with Lg. Following harvest, F_1_ plants were self-pollinated to generate the F_2_ generation. The parental lines and F_2_ segregating population were cultivated at the experimental farm of Anhui University of Science and Technology. The parental lines were planted in three rows each, while the F_2_ population was planted in 100 rows, with 10 plants per row. Row spacing was set at 0.5 m and plant spacing at 0.3 m. The experimental period was from 2022 to 2024.

### 2.2. BSA Sequencing and Data Analysis

In this study, sequencing was performed using the F_2_ population. The calculation of heritability followed the method described by Wang et al. [20]. Small-grain pool and large-grain pool were constructed based on the distribution analysis of individual plants in the 402-individual F_2_ population. This study used the mean values of the small-grain parent (Sg) and large-grain parent (Lg) as reference lines for classification within the F_2_ population, with 31 samples from the small-grain bulk and 15 samples from the large-grain bulk analyzed. High-quality DNA was extracted from selected plants using the DNAsecure Plant Genomic DNA Extraction Kit (Tiangen, Beijing, China). Then DNA content of every sample was measured by Qubit 4 (Thermo Fisher Scientific, Waltham, MA, USA). All samples were diluted to 30 μg/μL and mixed to form the sequencing pool. Two pools and the parents were sent to BGI for sequencing using DNBSEQ-T7 (MGI, Wuhan, China). The sequencing depth for every pool was 30× coverage. Raw sequencing data were filtered by FastQC. Clean reads were mapped to Sorghum bicolor v5.1. SNPs were called by samtools 1.10 using default parameters. Then SNP index was calculated by QTL-seq [21]. Confidence interval of 95% and 99% was used as thresholds to screen candidate intervals and loci.

### 2.3. Molecular Marker Design and Genetic Map Construction

Molecular markers were designed based on the results of BSA analysis. InDel loci with a sequencing depth of ≥30 and length difference ≥ 6 were selected to design primers using Primer5 with amplified size between 90–130 bp. The designed primers were synthesized by Sangon Biotech Company (Nanjing, China). The primers were listed in Table S1. The fine-mapping experiment utilized 457 recombinant individuals derived from a newly established F_2_ population.

PCR reaction mixture was prepared by combining 1.5 μL of primers (1 μM), 1.5 μL of template DNA (20–30 ng/μL), 5 μL of 2×Magic Green Taq SuperMix (ToloBio, Chuzhou,, China), and 2 μL of ddH_2_O. PCR was performed with an ETC811 thermal cycler (Eastwin, Beijing, China) using 94 °C for 5 min, followed by 38 cycles of 94 °C for 30 s, 58 °C for 30 s, and 72 °C for 1 min, before 72 °C for 10 min and 4 °C.

Polyacrylamide Gel Electrophoresis (PAGE) [22] was used for the separation of primer-amplified sequences in an 8% acrylamide gel at 220 V. After electrophoresis, the gel was stained with a silver solution.

Mapmarker 3.0 [23] was used to calculate the genetic distance between markers and loci based on the Indel markers. Bands consistent with the Sg parent were coded as “0”, those consistent with Lg were coded as “2”, and heterozygous results were coded as “1”. Genetic distances were used to construct genetic maps with Mapdraw [24], perform linkage analysis of molecular markers, and screen QTL candidate intervals.

### 2.4. RNA-Seq Analysis

RNA was extracted from panicles of Sg and Lg at 0 and 14 days after heading (DAH) using an RNA extraction kit (Tiangen, China), with three biological replicates. The quality and quantity of RNA were detected using an Agilent 2100 bioanalyzer (Agilent, Santa Clara, CA, USA). Samples were sent to BGI (NovaSeq 6000; Illumina, Wuhan, China) for library construction and sequencing [25]. Raw data of RNA sequencing were filtered using FastQC. Hisat2 was used to map reads to the reference genome (*Sorghum bicolor* v5.1).

DESeq2 [26] was used to identify significant expression differences of genes between the two samples. Genes with |log_2_FoldChange| ≥ 1 and *p*_adj_ ≤ 0.05 were selected as differentially expressed genes (DEGs). Venn diagram analysis was performed on DEGs from two comparative groups to display common DEGs. GO and KEGG enrichment analyses were conducted using clusterProfiler 4.0 [27].

### 2.5. Quantitative PCR (qPCR)

qPCR was used to validate candidate gene expression. qPCR primers were designed using the primer design website https://quantprime.mpimp-golm.mpg.de/ (accessed on 25 December 2024). Total RNA was extracted from day 0 using the RNAprep Pure Plant RNA Extraction Kit (TIANGEN, China). cDNA was synthesized from 1 μg RNA using the ToloScript All-in-one RT EasyMix for qPCR (ToloBio, Chuzhou, China). qPCR was performed using Applied Biosystems equipment (Thermo Fisher Scientific, Suzhou, China). *Ubiquitin* was used as a reference gene [28]. The relative gene expression level was analyzed using the 2^−ΔΔCT^ method [29]. Three biological replicates were used in qPCR.

### 2.6. Statistical Analysis

Grain length, grain width, and 1000-grain weight were measured for the parental lines. For the F_2_ population, 1000-grain weight was measured for each individual plant. T-tests and distribution analyses were conducted using Excel 2016. Normality tests were performed using GraphPad [30].

## 3. Results

### 3.1. Grain Phenotypic Analysis of Parents

**Figure 1:**
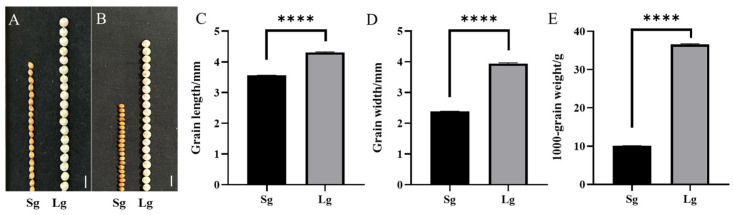
Comparison and statistical analysis of grain traits between sorghum accessions Sg and Lg. Comparison of 20-grain length between Sg and Lg, bar = 4 mm (**A**), 20-grain width between Sg and Lg, bar = 4 mm (**B**), statistic analysis of grain length between Sg and Lg (**C**), grain width between Sg and Lg (**D**), and 1000-grain weight between Sg and Lg (**E**), **** denotes *p* < 0.01.

The two accessions, Lg and Sg, showed significant differences in grain size (Figure 1A,B). The grain length of Sg and Lg was 3.56 ± 0.01 mm and 4.30 ± 0.03 mm, respectively, with the grain length of Lg being 1.22-fold greater than that of Sg. The grain width of Sg was 2.39 ± 0.01 mm, whereas that of Lg was 3.94 ± 0.03 mm, representing a 1.65-fold increase relative to Sg. (Table S2). The grain length of Lg was about 14 mm higher than Sg, and the grain width was about 21 mm higher than Sg. It means the grain length and width of Lg were about 20% and 40% higher than Sg, respectively (Figure 1A,B). Statistical analysis showed that there were significant differences between the two accessions in grain length, grain width, and 1000-grain weight (Figure 1C–E).

The 1000-grain weight of Sg was 10.07 ± 0.03 g, while Lg was 36.56 ± 0.14 g, i.e., the 1000-grain weight of Lg was 3.63 times that of Sg. F_1_ plants showed a similar phenotype to Lg in the three traits (Figure S1). Therefore, it showed that the large grain size of sorghum is a dominant trait in this study.

### 3.2. Distribution of 1000-Grain Weight in the F_2_ Segregating Population

**Figure 2:**
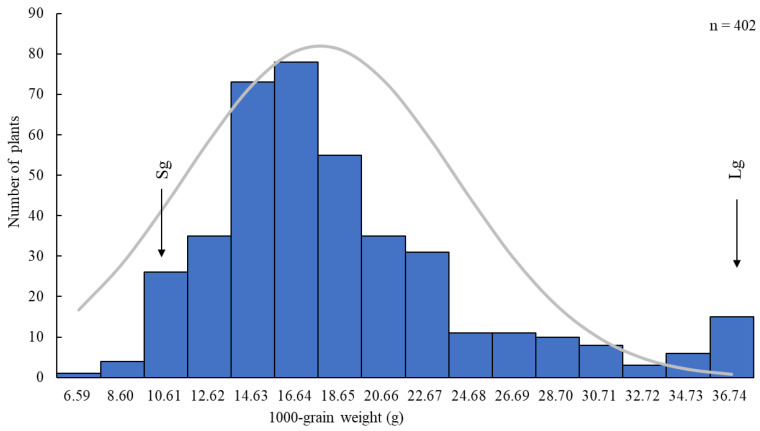
Frequency distribution of the 1000-grain weight in the Lg (large-grain) × Sg (small-grain) F_2_ population.

Among the grain size-related traits, the 1000-grain weight showed the highest difference and was used as the main trait for genetic population distribution analysis. In the F_2_ population, the 1000-grain weight of 402 individual plants displayed a normal distribution (Figure 2). This study used the mean values of the small-grain parent (Sg) and large-grain parent (Lg) as reference lines for classification within the F_2_ population. A total of 31 individuals exhibited 1000-grain weights similar to that of the small-grain parent Sg, while 15 individuals exhibited values close to that of the large-grain parent Lg. The heritability of the 1000-grain weight is 44.36%. These 31 and 15 individuals were subsequently used to construct the small-grain and large-grain sequencing pools, respectively, for BSA-seq analysis and genetic map construction.

### 3.3. BSA Sequencing Analysis and Fine Mapping of qGS1

**Figure 3:**
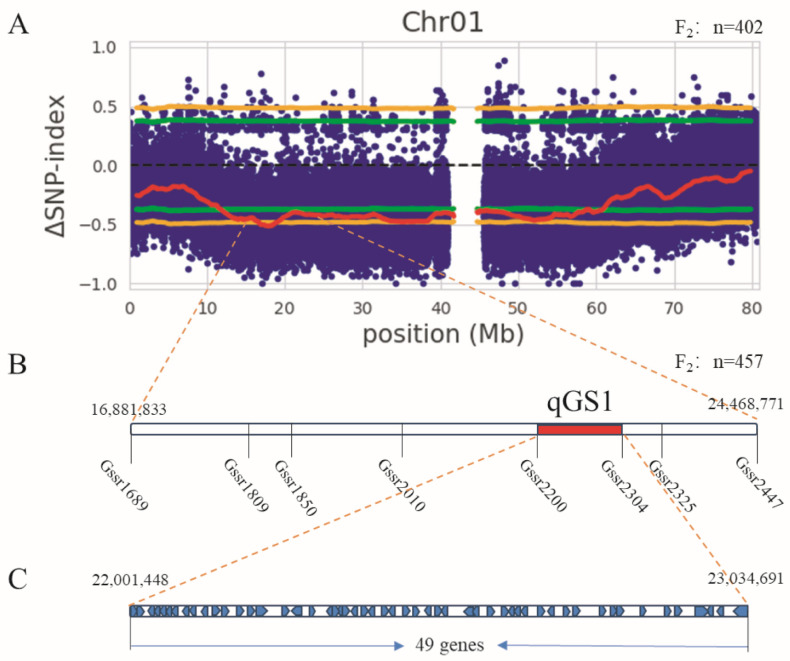
Mapping of grain size genes. (**A**) SNP index on Chr01. The x-axis represents the sorghum chromosome size (Mb), while the y-axis represents the delta SNP-index. The red line is the delta SNP-index curve, the green line is the threshold for the 95% confidence interval, and the yellow line is the threshold for the 99% confidence interval. (**B**) Linkage analysis of polymorphic primers, unit: cM. (**C**) Candidate genes of *qGS1*.

The BSA sequencing results were analyzed using SNP-index [21] for linkage region analysis (Figure S2). The genotypic frequencies of the F_2_ population at specific SNP loci were analyzed based on SNP differences. According to the ΔSNP-index analysis, when the 95% confidence interval was used, SNP differences exceeded the threshold on multiple chromosomes, with QTL regions for grain weight distributed at 12–61 Mb on chromosome 1 and 21–50 Mb on chromosome 3. Due to the large candidate intervals, further screening of the QTL regions was performed. When the 99% confidence interval was applied (Figure 3A), only SNPs on chromosome 1 exceeded the top 99% threshold, while those on chromosome 3 did not reach the 99% selection effect threshold, thus, preliminarily indicating that the major grain size gene *qGS1* for sorghum is likely located on chromosome 1.

Due to the large QTL interval on chromosome 1, SSR molecular marker primers were designed within the QTL interval. Selective genotyping analysis was performed using 55 extremely small-grain recombinant individual plants for typing statistics. A total of 48 pairs of primers were designed, with an SSR molecular marker primer polymorphism rate of 33%, among which 8 pairs of polymorphic primers were closely associated with grain size traits (Table S1). According to the results shown in the genetic map, *qGS1* is located between the Gssr2200 and Gssr2304 markers, with a genetic distance of 23.5 cM for *qGS1*, an LOD value of 3. This analysis preliminarily mapped *qGS1* to the interval Chr01: 22,001,448–23,035,593 (approximately 1 Mb) on chromosome 1 (Figure 3B). For the genomic region identified by genetic mapping, 49 candidate genes for grain weight gene *qGS1* were screened by integrating annotation files (Figure 3C, Table S3).

### 3.4. Transcriptome Analysis

To elucidate the molecular mechanisms underlying grain size regulation, transcriptome analysis was conducted on spike materials of Sg and Lg at 0 and 14 DAH. Comparative analysis of 12 transcriptome sequencing results (Table S4) showed that clean reads averaged 21.82 million, with a mean mapping rate exceeding 91% and Q20 values above 97%, indicating high-quality and reliable sequencing data. Clustering analysis (Figure S3) revealed strong correlations and high consistency among biological replicates of the same plant variety at the same heading time.

**Figure 4:**
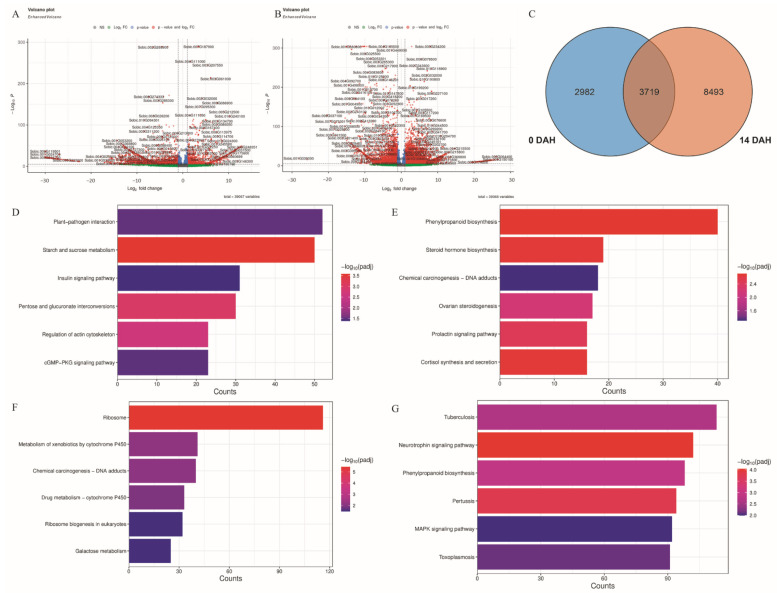
Differential gene expression and pathway enrichment analyses. (**A**) Volcano plot of differentially expressed genes (DEGs) between Sg and Lg at 0 days after heading (DAH). (**B**) Volcano plot of DEGs between Sg and Lg at 14 DAH. (**C**) Venn diagram showing the number of DEGs identified at 0 and 14 DAH, including their overlap. (**D**) KEGG pathway enrichment analysis of downregulated DEGs at 0 DAH. (**E**) KEGG pathway enrichment analysis of upregulated DEGs at 0 DAH. (**F**) KEGG pathway enrichment analysis of downregulated DEGs at 14 d DAH. (**G**) KEGG pathway enrichment analysis of upregulated DEGs at 14 DAH.

Differentially expressed genes (DEGs) were visualized using volcano plots. At 0 DAH (Figure 4A), a higher density of red dots on the left indicated a predominance of significantly downregulated genes in Sg vs. Lg. Conversely, at 14 DAH (Figure 4B), a greater density of red dots on the right indicated a predominance of significantly upregulated genes. A total of 6701 and 12,212 DEGs were identified at 0 and 14 DAH, respectively, with 3719 DEGs commonly present across both time points (Figure 4C, Table S5). At 0 DAH, upregulated DEGs were enriched in 6 Gene Ontology (GO) terms. In the cellular component (CC) category, 5 terms were significantly enriched, with “cell wall” and “external encapsulating structure” containing the most genes (52 DEGs) (Figure S4, Table S6). Downregulated DEGs were enriched in 137 GO terms, including 104 terms in the biological process (BP) category; the terms “inorganic molecular entity transmembrane transporter activity” and “homeostatic process” had the highest gene counts (86 DEGs) (Figure S4, Table S6). At 14 DAH, upregulated DEGs were enriched in 151 GO terms, with 76 terms in the BP category; “cell wall and external encapsulating structure” again had the most genes (151 DEGs) (Figure S4, Table S6). Downregulated DEGs were enriched in 41 GO terms, with 31 terms in the BP category, where “nucleolus” included the most genes (120 DEGs) (Figure S4, Table S6). The functional categories with the highest enrichment of upregulated DEGs were consistent between 0 and 14 DAH.

KEGG pathway enrichment analysis of upregulated DEGs at 0 DAH identified 12 significantly enriched pathways, including “Phenylpropanoid biosynthesis” (40 DEGs), “Steroid hormone biosynthesis” (19 DEGs), and “Chemical carcinogenesis-DNA adducts” (18 DEGs) (Figure 4D, Table S8). Downregulated DEGs were enriched in 9 pathways, primarily in “Starch and sucrose metabolism” (50 DEGs), “Plant-pathogen interaction” (52 DEGs), and “Insulin signaling pathway” (31 DEGs) (Figure 4E, Table S7). At 14 DAH, upregulated DEGs were enriched in 41 pathways, with the top categories being “Tuberculosis” (113 DEGs), “Neurotrophin signaling pathway” (102 DEGs), and “Phenylpropanoid biosynthesis” (98 DEGs) (Figure 4F, Table S8). In contrast, downregulated DEGs were enriched in 7 pathways, notably “Ribosome” (116 DEGs), “Metabolism of xenobiotics by cytochrome P450” (41 DEGs), and “Chemical carcinogenesis-DNA adducts” (40 DEGs) (Figure 4G, Table S7). These findings suggest stage-specific molecular pathways are involved in grain size regulation, with “Starch and sucrose metabolism” downregulated at heading and “Phenylpropanoid biosynthesis” upregulated during grain filling likely playing important regulatory roles.

### 3.5. Screening of qGS1 Candidate Genes by Integrating BSA-Seq and RNA-Seq Analyses

To identify key genes regulating grain size within the *qGS1* locus, a combined analysis of the *qGS1* interval mapping and RNA sequencing (RNA-seq) data was performed. Genes located within the finely mapped *qGS1* interval were intersected with differentially expressed genes (DEGs) identified at 0 and 14 DAH. Ten DEGs were detected at 0 DAH, and fifteen DEGs were detected at 14 DAH. This integrative approach revealed six genes that were significantly differentially expressed at both developmental stages (Table 1). Among RNA-Seq analyses, five genes—*Sobic.001G229100*, *Sobic.001G229401*, *Sobic.001G229850*, *Sobic.001G230700*, and *Sobic.001G233200*—were consistently downregulated in the Sg genotype compared with the Lg genotype, while *Sobic.001G233300* was upregulated. (Figure S5). qPCR results were consistent with the results of RNA-seq.

**Figure 5:**
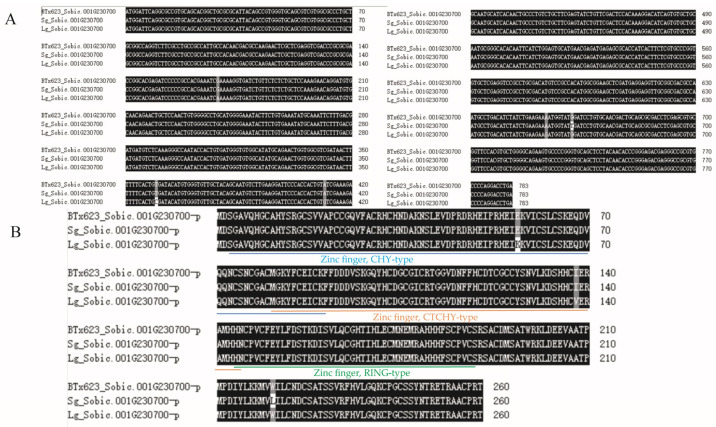
The sequence analysis of *Sobic.001G230700*. (**A**) Comparative analysis of nucleotide sequences (CDS region) was conducted for *Sobic.001G230700* across three genotypes: BTx623, Sg, and Lg. (**B**) Comparative analysis of protein sequences was conducted for *Sobic.001G230700* across three genotypes: BTx623, Sg, and Lg. Zinc finger, CHY-type: 3–92. Zinc finger, CTCHY-type: 81–145. Zinc finger, RING-type: 144–189.

Functional annotation suggested that several of these genes were involved in critical molecular pathways. Notably, ***Sobic.001G230700*** encodes a zinc finger protein involved in transcriptional regulation and is linked to the “RCHY1/PIRH2” pathway. The remaining genes either encode uncharacterized proteins or zinc-binding proteins with putative roles in reverse transcription. Comparative analysis of nucleotide sequences was conducted for *Sobic.001G230700* across three genotypes: BTx623, Sg, and Lg. In the coding sequence (CDS) region of *Sobic.001G230700*, one and four nucleotide substitutions were identified in Sg and Lg relative to BTx623, respectively, resulting in one and two amino acid changes (Figure 5). Amino acid modifications in the protein sequence are annotated as EEK at residue 57, IIV at residue 138, and WLW at residue 221. EEK is embedded within the CHY-type zinc finger domain, while IIV localizes to the CTCHY-type zinc finger domain. The 1000-grain weight of BTx623 was 25.38 ± 0.42 g, with a closer proximity to the Lg material. *Sobic.001G230700* is a strong candidate for the *qGS1* gene.

## 4. Discussion

This study utilized Sg and Lg as research materials to map new grain size QTL loci. Due to external factors such as time and climate, a selective genotyping analysis method was employed, which has been validated by previous studies as time-saving, labor-efficient, and feasible [31,32,33]. In this study, only 55 recessive extreme individual plants were used for preliminary mapping, similar to Han et al. [14], who selected 47 extreme large-grain and 47 extreme small-grain individuals for QTL analysis of sorghum grain weight. However, because the grain size QTL on chromosome 1 spanned a large range (approximately 49 Mb), selective genotyping analysis was used to narrow the genetic distance interval of *qGS1* to 0–23.5 cM. However, Charcosset et al. [34] reported that reducing the QTL mapping population size decreases QTL detection power and increases QTL confidence intervals, which is consistent with the results of this study. The *qGS1* identified in this study is located on chromosome 1 at positions 22,001,448–23,035,593. Chiluwal et al. [35] identified and analyzed three QTLs associated with grain yield during grain filling, among which the QTL located on chromosome 1 (52.23–61.18 Mb) showed no positional overlap with the results of this study. Tao et al. [36] conducted a detailed genome-wide association study (GWAS) on grain traits in sorghum and identified multiple QTLs related to grain size. Among the GWAS results, qGS1.7 (Chr01: 21,347,457–21,451,374) is the closest to the position of qGS1 reported herein but does not overlap with it. Thus, qGS1 is a new locus controlling seed size in sorghum.

Grain size and weight in cereal crops are regulated by multiple signaling pathways, such as the ubiquitin-proteasome, G protein, MAPK signaling, and phytohormone signaling pathways. The zinc-finger protein *Sobic.001G230700* is involved in the “RCHY1/PIRH2” pathways. SlCHYR1, a protein containing RING and CHY zinc finger domains, promotes tomato fruit ripening by reprogramming abscisic acid and ethylene signaling [37]. The C3H15 protein negatively regulates cell elongation by inhibiting the “brassinosteroid (BR) signaling” pathway, thereby affecting organ size and grain development [38]. The maize C4HC3-type zinc-finger protein gene *ZmZFP2* influences grain size by regulating endosperm cell proliferation and starch synthesis [39]. The zinc-finger protein encoded by the *TaABI3-A1* gene regulates the synthesis of starch and storage proteins in wheat endosperm, impacting wheat grain size and quality [5]. Furthermore, Sorghum GeneAtlas v2 FPKM [40] indicates that Sobic.001G230700 has the highest expression at seed imbibed.grain maturity stage (Table S8). The study by Enyew et al. [41] demonstrated the influence of SNPs in the *Sobic.001G230700* locus on flowering time. Therefore, *Sobic.001G230700* is proposed as a candidate gene for the sorghum grain size locus *qGS1*.

Combining BSA-seq and RNA-Seq to screen candidate genes is a commonly used method to deeply understand the genetic basis of grain weight and mine related favorable alleles. Using a combined BSA-Seq and RNA-Seq analysis approach, Wang et al. [42] revealed the mapping and identification of candidate genes for cadmium accumulation in *Brassica napus*; Gao et al. [43] uncovered molecular pathways and genes related to plant height in foxtail millet (*Setaria italica*); Yang et al. [44] analyzed and identified candidate genes for seed weight in *Brassica juncea*. However, reports on the combined application of BSA and RNA-seq in sorghum grain gene research remain scarce. In this study, an F_2_ segregating population obtained by hybridizing and selfing sorghum materials Sg and Lg was used as the research material to analyze the genetic characteristics of offspring traits. Extreme trait pools were constructed for BSA sequencing analysis, and combined with RNA-Seq analysis during the sorghum grain filling stage, two grain size-related candidate genes were identified. Based on the exploratory results of this study, the combined analysis of BSA-seq and RNA-seq provides an efficient pathway to clone key genes and elucidate the molecular basis of crop yield formation.

## Figures and Tables

**Table 1:** Down-/up-regulated differentially expressed genes (DEGs) a t0 and 14 DAH within the *qGS1* mapping interval.

Gene Number	Location	Function Annotation	Sg and Lg at 0 DAH	Sg and Lg at 14 DAH	KEGG
*Sobic.001G229100*	Chr01: 22,212,463–22,214,140	Acetylglucosaminyltransferase EXT1/exostosin 1	Down	Down	-
*Sobic.001G229401*	Chr01: 22,286,190–22,287,167	-	Down	Down	-
*Sobic.001G229850*	Chr01: 22,375,134–22,381,551	DNA helicase PIF1/RRM3	Down	Down	-
*Sobic.001G230700*	Chr01: 22,460,974–22,464,834	Zn-finger protein	Down	Down	RCHY1/PIRH2
*Sobic.001G233200*	Chr01: 22,987,418–22,988,410	zinc-binding in reverse transcriptase	Down	Down	-
*Sobic.001G233300*	Chr01: 22,989,390–22,990,975	-	Up	Up	-

## Data Availability

The original contributions presented in the study are included in the article/Supplementary Material. Further inquiries can be directed to the corresponding authors.

## Supplementary Materials

The following supporting information can be downloaded at: https://www.mdpi.com/article/10.3390/plants14121791/s1, Figure S1: Comparative statistical analysis of grain characteristics between F1-generation materials and parental materials; Figure S2: Chromosome linkage region analysis map; Figure S3: Heatmap of inter-sample correlation; Figure S4: GO enrichment analysis; Figure S5: qPCR of candidate genes; Table S1: Primers uesd in the article; Table S2: Statistics of grain-related traits of Sg and Lg; Table S3; Annotation table of grain size candidate genes; Table S4: Statistical Table of Transcriptome Sequencing Results; Table S5: Analysis of DEGs at 0 DAH and 14 DAH; Table S6: GO enrichment analysis data; Table S7: KEGG enrichment analysis data; Table S8: The expression of *Sobic.001G230700* in Sorghum GeneAtlas v2 FPKM.

## References

[1] McGinnis M.J., Painter J.E. (2020). Sorghum: History, use, and health benefits. Nutr. Today.

[2] Kalpande H., Surashe S., Badigannavar A., More A., Ganapathi T. (2022). Induced variability and assessment of mutagenic effectiveness and efficiency in sorghum genotypes [*Sorghum bicolor* (L.) Moench]. Int. J. Radiat. Biol..

[3] Abah C., Ishiwu C., Obiegbuna J., Oladejo A. (2020). Sorghum grains: Nutritional composition, functional properties and its food applications. Eur. J. Nutr. Food Saf..

[4] Figueiredo D.D., Köhler C. (2018). Auxin: A molecular trigger of seed development. Genes Dev..

[5] Zhao L., Chen J., Zhang Z., Wu W., Lin X., Gao M., Yang Y., Zhao P., Yao Y., Zhang A. (2024). Unraveling wheat endosperm development: Epigenetic regulation and novel regulators for enhanced yield and quality. bioRxiv.

[6] Huang H., Xie S., Xiao Q., Wei B., Zheng L., Wang Y., Cao Y., Zhang X., Long T., Li Y. (2016). Sucrose and ABA regulate starch biosynthesis in maize through a novel transcription factor, ZmEREB156. Sci. Rep..

[7] Ma B., Zhang L., He Z. (2023). Understanding the regulation of cereal grain filling: The way forward. J. Integr. Plant Biol..

[8] Xiao W., Brown R.C., Lemmon B.E., Harada J.J., Goldberg R.B., Fischer R.L. (2006). Regulation of seed size by hypomethylation of maternal and paternal genomes. Plant Physiol..

[9] Ji Y., Hewavithana T., Sharpe A.G., Jin L. (2024). Understanding grain development in the Poaceae family by comparing conserved and distinctive pathways through omics studies in wheat and maize. Front. Plant Sci..

[10] Baye W., Xie Q., Xie P. (2022). Genetic architecture of grain yield-related traits in sorghum and maize. Int. J. Mol. Sci..

[11] Tao Y., Mace E., George-Jaeggli B., Hunt C., Cruickshank A., Henzell R., Jordan D. (2018). Novel grain weight loci revealed in a cross between cultivated and wild sorghum. Plant Genome.

[12] Cao N., Ding Y., Xu J., Cheng B., Gao X., Li W., Zou G., Zhang L. (2024). QTL analysis of sorghum grain traits based on high-density genetic map. J. Appl. Genet..

[13] Zou G., Zhai G., Yan S., Li S., Zhou L., Ding Y., Liu H., Zhang Z., Zou J., Zhang L. (2020). Sorghum qTGW1a encodes a G-protein subunit and acts as a negative regulator of grain size. J. Exp. Bot..

[14] Han L., Chen J., Mace E.S., Liu Y., Zhu M., Yuyama N., Jordan D.R., Cai H. (2015). Fine mapping of qGW1, a major QTL for grain weight in sorghum. Theor. Appl. Genet..

[15] Tao Y., Luo H., Xu J., Cruickshank A., Zhao X., Teng F., Hathorn A., Wu X., Liu Y., Shatte T. (2021). Extensive variation within the pan-genome of cultivated and wild sorghum. Nat. Plants.

[16] Zhang D., Tang S., Liu F., Zhao K., Li C., Xia R., Yu F., Xie Q., Xie P. (2025). Natural variations in Multi-Grain Spikelet 1 enhance grain number in sorghum. J. Integr. Plant Biol..

[17] Zhu X., Gou Y., Heng Y., Ding W., Li Y., Zhou D., Li X., Liang C., Wu C., Wang H. (2023). Targeted manipulation of grain shape genes effectively improves outcrossing rate and hybrid seed production in rice. Plant Biotechnol. J..

[18] Wang G., Zhao Y., Mao W., Ma X., Su C. (2020). QTL analysis and fine mapping of a major QTL conferring kernel size in maize (*Zea mays*). Front. Genet..

[19] Belide S., Vanhercke T., Petrie J.R., Singh S.P. (2017). Robust genetic transformation of sorghum (*Sorghum bicolor* L.) using differentiating embryogenic callus induced from immature embryos. Plant Methods.

[20] Tang W., Huang L., Bu S., Zhang X., Wu W. (2018). Estimation of QTL heritability based on pooled sequencing data. Bioinformatics.

[21] Takagi H., Abe A., Yoshida K., Kosugi S., Natsume S., Mitsuoka C., Uemura A., Utsushi H., Tamiru M., Takuno S. (2013). QTL-seq: Rapid mapping of quantitative trait loci in rice by whole genome resequencing of DNA from two bulked populations. Plant J..

[22] Sambrook J., David W. (2001). Molecular Cloning: A Laboratory Manual.

[23] Lander E.S., Green P., Abrahamson J., Barlow A., Daly M.J., Lincoln S.E., Newberg L.A. (2008). Corrigendum to “MAPMAKER: An interactive computer package for constructing primary genetic linkage maps of experimental and natural populations” [Genomics 1 (1987) 174–181]. Genomics.

[24] Liu R.H., Meng J. (2003). MapDraw: A microsoft excel macro for drawing genetic linkage maps based on given genetic linkage data. Yi Chuan = Hered..

[25] Modi A., Vai S., Caramelli D., Lari M. (2021). The Illumina sequencing protocol and the NovaSeq 6000 system. Bacterial Pangenomics: Methods and Protocols.

[26] Love M.I., Huber W., Anders S. (2014). Moderated estimation of fold change and dispersion for RNA-seq data with DESeq2. Genome Biol..

[27] Wu T., Hu E., Xu S., Chen M., Guo P., Dai Z., Feng T., Zhou L., Tang W., Zhan L. (2021). clusterProfiler 4.0: A universal enrichment tool for interpreting omics data. Innovation.

[28] Sudhakar Reddy P., Srinivas Reddy D., Sivasakthi K., Bhatnagar-Mathur P., Vadez V., Sharma K.K. (2016). Evaluation of sorghum [*Sorghum bicolor* (L.)] reference genes in various tissues and under abiotic stress conditions for quantitative real-time PCR data normalization. Front. Plant Sci..

[29] Livak K.J., Schmittgen T.D. (2001). Analysis of relative gene expression data using real-time quantitative PCR and the 2^−ΔΔCT^ method. Methods.

[30] Swift M.L. (1997). GraphPad prism, data analysis, and scientific graphing. J. Chem. Inf. Comput. Sci..

[31] Darvasi A., Soller M. (1992). Selective genotyping for determination of linkage between a marker locus and a quantitative trait locus. Theor. Appl. Genet..

[32] Phansak P., Soonsuwon W., Hyten D.L., Song Q., Cregan P.B., Graef G.L., Specht J.E. (2016). Multi-population selective genotyping to identify soybean [Glycine max (L.) Merr.] seed protein and oil QTLs. G3 Genes Genomes Genet..

[33] Ćeran M., Đorđević V., Miladinović J., Vasiljević M., Đukić V., Ranđelović P., Jaćimović S. (2024). Selective Genotyping and Phenotyping for Optimization of Genomic Prediction Models for Populations with Different Diversity. Plants.

[34] Charcosset A., Gallais A. (1996). Estimation of the contribution of quantitative trait loci (QTL) to the variance of a quantitative trait by means of genetic markers. Theor. Appl. Genet..

[35] Anuj C., Ramasamy P., Poudel H.P., Kebede M., Troy O., Lauren F., Meghnath P., Bean S.R., Sebela D., Raju B. (2022). Genetic control of source–sink relationships in grain sorghum. Planta.

[36] Tao Y., Zhao X., Wang X., Hathorn A., Hunt C., Cruickshank A.W., van Oosterom E.J., Godwin I.D., Mace E.S., Jordan D.R. (2020). Large-scale GWAS in sorghum reveals common genetic control of grain size among cereals. Plant Biotechnol. J..

[37] Cheng F., Huang J., Tang P., Li Y., Hu Z., Cui B., Xie X., Chen Q., Tian J., Gu H. (2022). SlCHYR1, a RING and CHY zinc finger domain-containing protein, promotes tomato fruit ripening by reprograming abscisic acid and ethylene signaling. Sci. Hortic..

[38] Chai G., Qi G., Wang D., Zhuang Y., Xu H., Bai Z., Bai M.-Y., Hu R., Wang Z.-y., Zhou G. (2022). The CCCH zinc finger protein C3H15 negatively regulates cell elongation by inhibiting brassinosteroid signaling. Plant Physiol..

[39] Zhang L., Wang Q., Li W., Zheng Q., Fu M., Wang H., Li X., Wang Y., Hu L., Yao W. (2025). ZmZFP2 encoding a C4HC3-type RING zinc finger protein regulates kernel size and weight in maize. Crop J..

[40] McCormick R.F., Truong S.K., Sreedasyam A., Jenkins J., Shu S., Sims D., Kennedy M., Amirebrahimi M., Weers B.D., McKinley B. (2018). The Sorghum bicolor reference genome: Improved assembly, gene annotations, a transcriptome atlas, and signatures of genome organization. Plant J..

[41] Enyew M., Feyissa T., Carlsson A.S., Tesfaye K., Hammenhag C., Seyoum A., Geleta M. (2022). Genome-wide analyses using multi-locus models revealed marker-trait associations for major agronomic traits in Sorghum bicolor. Front. Plant Sci..

[42] Wang H., Liu J., Huang J., Xiao Q., Hayward A., Li F., Gong Y., Liu Q., Ma M., Fu D. (2023). Mapping and Identifying Candidate Genes Enabling Cadmium Accumulation in Brassica napus Revealed by Combined BSA-Seq and RNA-Seq Analysis. Int. J. Mol. Sci..

[43] Gao Y., Yuan Y., Zhang X., Song H., Yang Q., Yang P., Gao X., Gao J., Feng B. (2022). Conuping BSA-Seq and RNA-Seq Reveal the Molecular Pathway and Genes Associated with the Plant Height of Foxtail Millet (Setaria italica). Int. J. Mol. Sci..

[44] Yang B., Yang L., Kang L., You L., Chen H., Xiao H., Qian L., Rao Y., Liu Z. (2024). Integrated analysis of BSA-seq and RNA-seq identified the candidate genes for seed weight in Brassica juncea. Front. Plant Sci..

