# Supplementary material for: Combined Analysis of BSA-Seq and RNA-Seq Reveals Candidate Genes for qGS1 Related to Sorghum Grain Size
Source: Plants (Basel). 2025 Jun 11;14(12):1791. doi: 10.3390/plants14121791 (PMC12196917; doi:10.3390/plants14121791)
Supplement: Supplementary file 1 [file plants-14-01791-s001.zip › Supplementary Files/Figure S1.pdf]

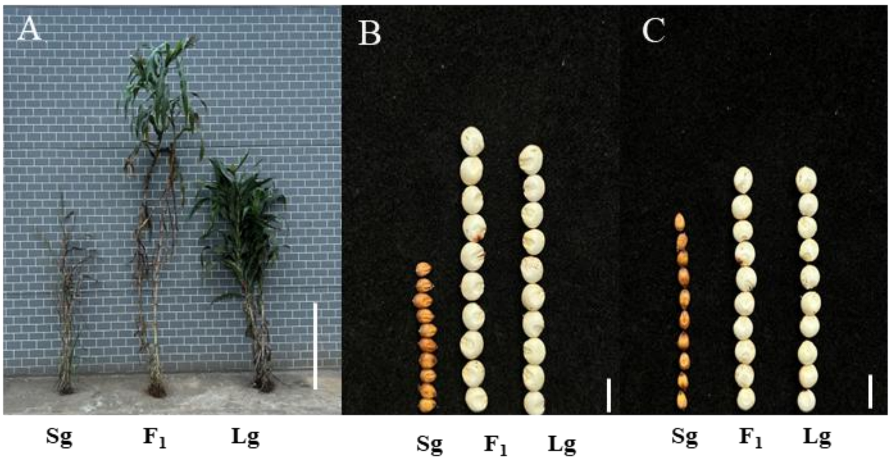

Figure S1 Comparative statistical analysis of grain characteristics between F_1-generation materials and parental materials. (A) visual comparison chart of plant type, bar = 1 m; (B) 10 grain width view, bar = 4 mm; (C) 10 grain length view, bar = 4 mm.
