# Supplementary material for: Combined Analysis of BSA-Seq and RNA-Seq Reveals Candidate Genes for qGS1 Related to Sorghum Grain Size
Source: Plants (Basel). 2025 Jun 11;14(12):1791. doi: 10.3390/plants14121791 (PMC12196917; doi:10.3390/plants14121791)
Supplement: Supplementary file 1 [file plants-14-01791-s001.zip › Supplementary Files/Figure S2.pdf]

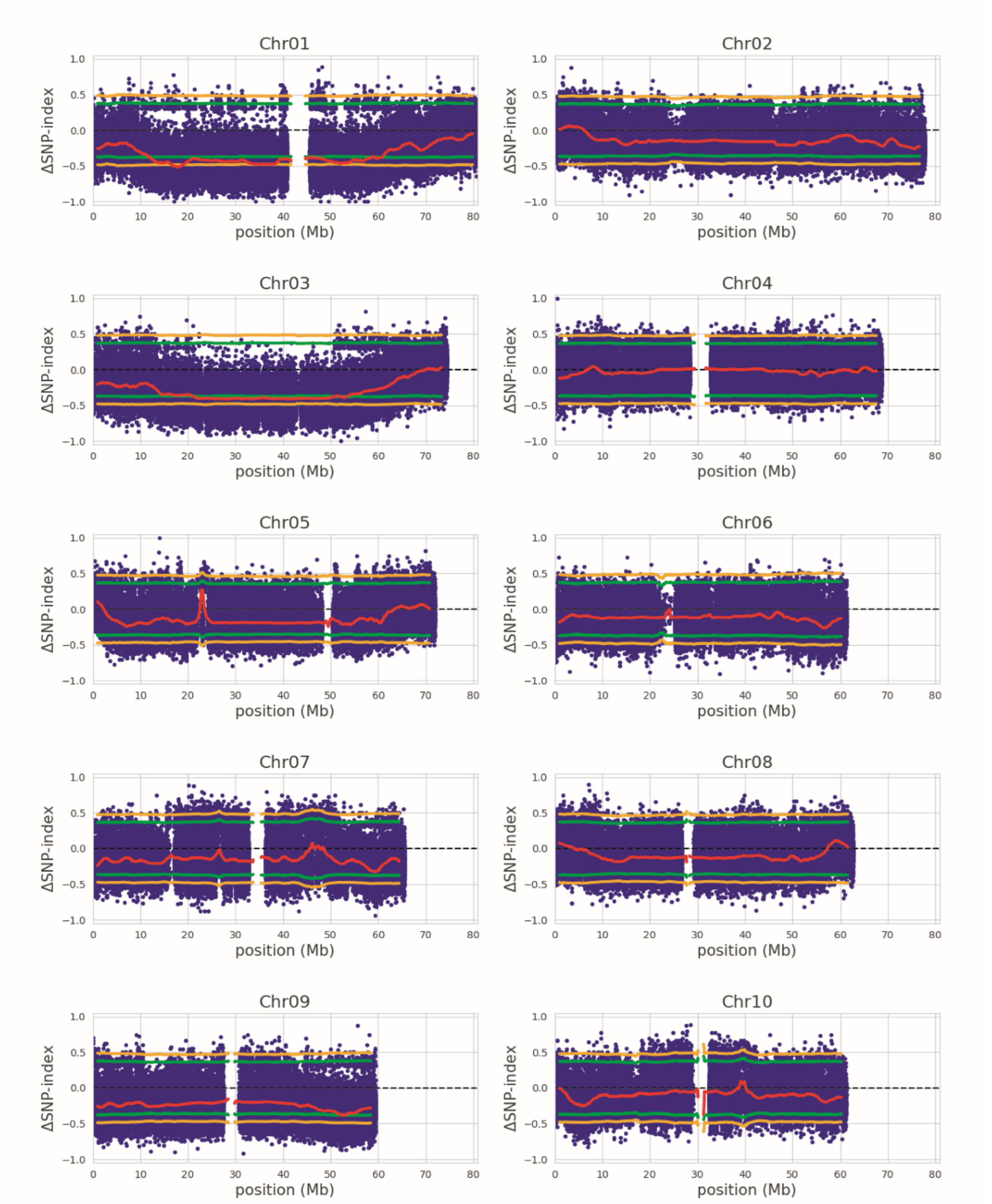

Figure S2 Chromosome linkage region analysis map. The x-axis represents the sorghum chromosome size (Mb), while the y-axis represents the delta SNP-index. The red line is the delta SNP-index curve, the green line is the threshold for the 95% confidence interval, and the yellow line is the threshold for the 99% confidence interval.
