# Supplementary material for: Combined Analysis of BSA-Seq and RNA-Seq Reveals Candidate Genes for qGS1 Related to Sorghum Grain Size
Source: Plants (Basel). 2025 Jun 11;14(12):1791. doi: 10.3390/plants14121791 (PMC12196917; doi:10.3390/plants14121791)
Supplement: Supplementary file 1 [file plants-14-01791-s001.zip › Supplementary Files/Figure S5.pdf]

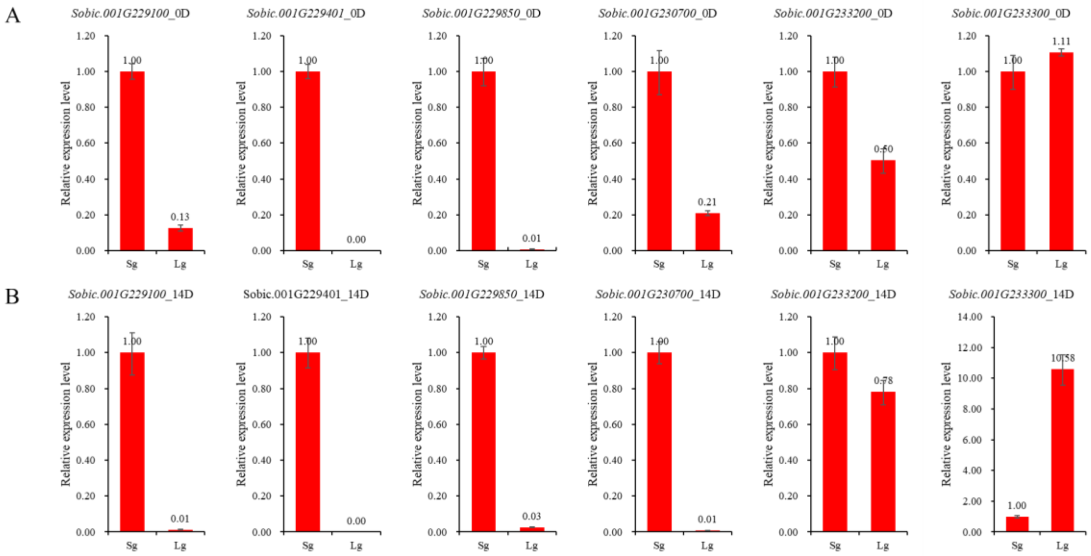

Figure S5 qPCR of candidate genes. (A) Relative expression of candidate genes in Sg and Lg at heading of 0 day; (B) Relative expression of candidate genes in Sg and Lg at heading of 14 day.
