# Supplementary material for: Combined Analysis of BSA-Seq and RNA-Seq Reveals Candidate Genes for qGS1 Related to Sorghum Grain Size
Source: Plants (Basel). 2025 Jun 11;14(12):1791. doi: 10.3390/plants14121791 (PMC12196917; doi:10.3390/plants14121791)
Supplement: Supplementary file 1 [file plants-14-01791-s001.zip › Supplementary Files/Figure S4.pdf]

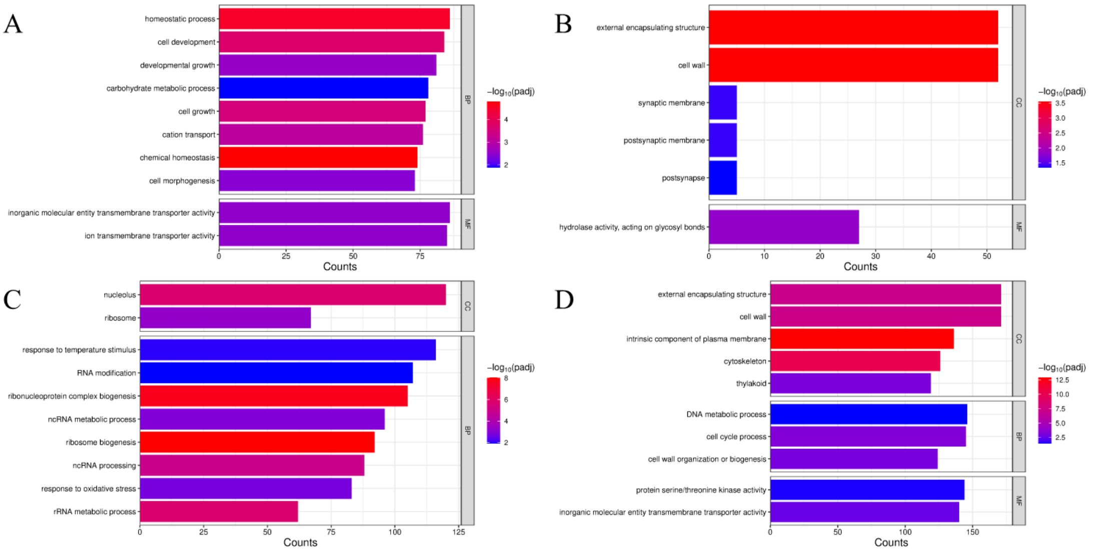

Figure S4 GO enrichment analysis. (A) GO enrichment analysis of down-regulated DEGs at heading of 0D; (B) GO enrichment analysis of down-regulated DEGs at heading of 14D; (C) GO enrichment analysis of up-regulated DEGs at heading of 0D; (D) GO enrichment analysis of down-regulated DEGs at heading of 14D.
