# Supplementary figures and images for: Combined Analysis of BSA-Seq and RNA-Seq Reveals Candidate Genes for qGS1 Related to Sorghum Grain Size
Source: Plants (Basel). 2025 Jun 11;14(12):1791. doi: 10.3390/plants14121791 (PMC12196917; doi:10.3390/plants14121791)

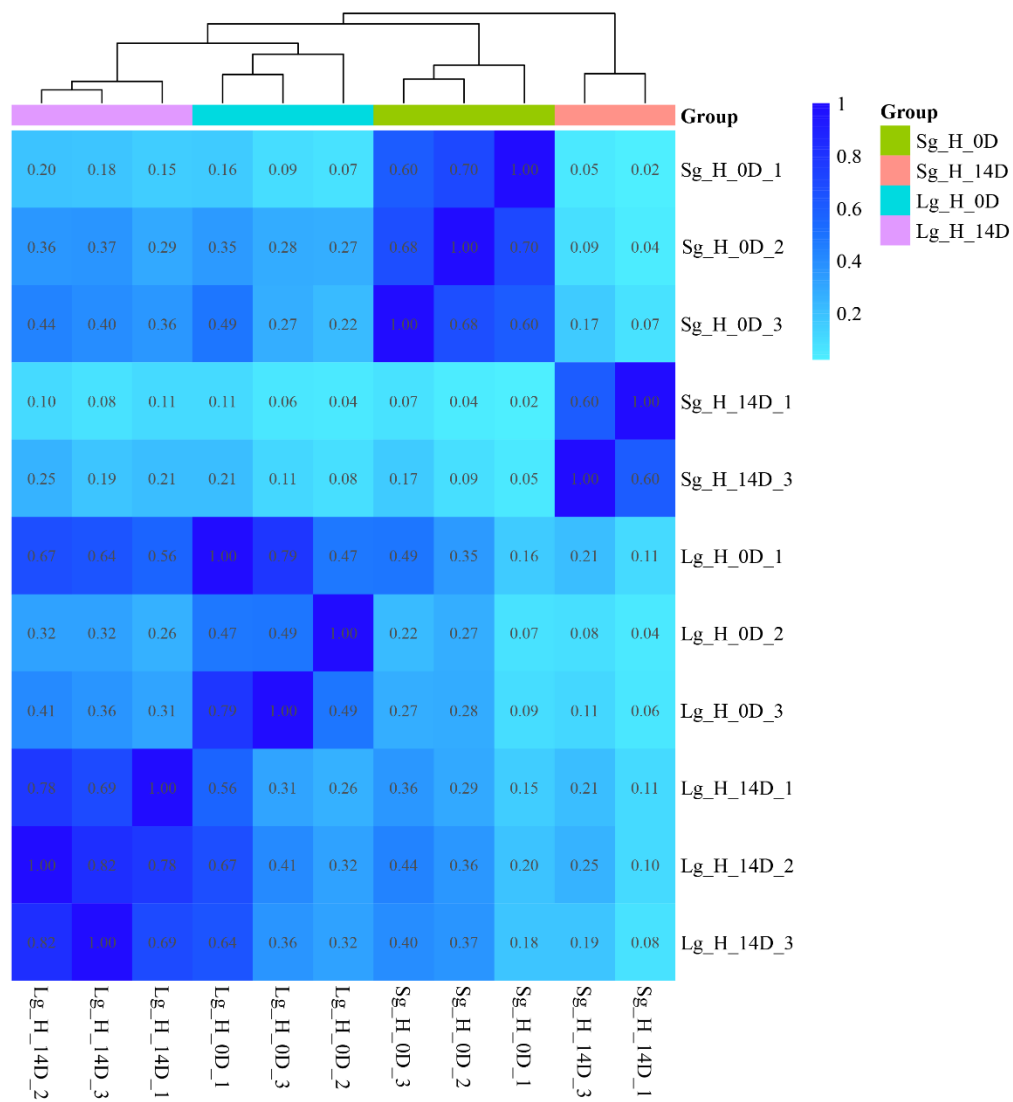

Figure S3 Heatmap of inter-sample correlation.

Supplement: Supplementary file 1 [file plants-14-01791-s001.zip › Supplementary Files/Figure S3.pdf]
